# Association between prostate-specific antigen change over time and prostate cancer recurrence risk: A joint model

**DOI:** 10.22088/cjim.11.3.324

**Published:** 2020-05

**Authors:** Reza Ali Mohammadpour, Ahad Alizadeh, Mohammad-Reza Barzegar, Abazar Akbarzadeh Pasha

**Affiliations:** 1 Department of Biostatistics, Faculty of Health, Health Sciences Research Center, Mazandaran University of Medical Sciences, Sari, Iran; 2 Student Research Committee, Faculty of Health, Mazandaran University of Medical Sciences, Sari, Iran; 3 Cancer Institute of Tehran, Tehran University of Medical Sciences, Tehran, Iran; 4 Department of Urology, Babol University of Medical Sciences, Babol, Iran

**Keywords:** Prostate specific antigen (PSA), Prostate cancer recurrence, Joint modeling

## Abstract

**Background:**

Prostate specific antigen (PSA) is an important biomarker to monitor patients after treated with radiation therapy (RT). The aim of this study is to evaluate the relationship between the PSA data and prostate cancer recurrence using the joint modeling.

**Methods:**

This historical cohort study was performed on 422 prostate cancer patients. Inclusion criteria included: patients with localized prostate cancer referring to Cancer Institute in Tehran (Iran) from 2007 to 2012, and under radiation therapy. Joint model has two components or sub-models. We showed the results by parameter estimating the longitudinal sub-model and survival sub-model. EM algorithm, Newton-Gauss and Gauss-Hermit law were used for final model parameters. R software version 3.2 was used for statistical analysis.

**Results:**

In this study, considering the inclusion and exclusion criteria, out of 422 patients, the data on 314 cases were selected for analysis and the main result of joint model was obtained. PSA directly and significantly was associated with recurrence risk, therefore increasing 2.6 ml/lit PSA (one unit in transformed PSA) increases 39% recurrence risk (95% CI for RR: 1.09-1.77). Also, slope of PSA trend has significant association with prostate cancer recurrence risk (95% CI for RR: 1.05-1.41).

**Conclusion:**

This study showed a significant relationship between PSA, and its slope with the recurrence risk by joint model, with regard to the pathological, demographic and clinical features in the Iranian population.

Prostate specific antigen (PSA) is an important biomarker to monitor patients post radiation therapy (RT) (1). It has been proven that prostate cancer (PC) tissue increases PSA 10 times higher in the patients’ blood serum (2). Therefore, increase of PSA concentration in blood indicates PC recurrence or metastasis (3, 4). After radiation therapy, rising of PSA level > 2 ng/mL above the nadir PSA, is the most reliable sign of persistent or recurrent disease (5). Patients were followed post radiation therapy over time and PSA in each visit was measured. Post treatment PSA and its whole trajectory over time and other baseline predictive variables or pre- treatment covariates are informative for detecting a clinical recurrence (6). Clinical and pathological characteristics such as; t-stage, Gleason score, type of treatments and age are confounding variables on the association between PSA change and PC recurrence risk. For the many reasons, the circumstance of this association is complex. First, PSA is an internal variable that is depended on mechanism of disease (7). Second, for everyone, PSA is measured many times and different frequencies. Third, PSA is measured with error. Forth, PSA has within and between groups variation. Fifth, recurrence time is not precise for some patients (censoring). Joint modeling is appropriate for these situations. Pauler Donna et al., studied the joint model for non-linear longitudinal, biomarker and time to the recurrence of the disease.

The Markof Chain Monte Carlo (MCMC) algorithm for parameter estimation was used (8). Yu et al., used the joint model for longitudinal, survival and cure on patients with prostate carcinoma (9). Taylor et al., used Bayesian estimation methods for joint model in predicting the risk of recurrence from longitudinal PSA measures (10, 11). Poust-Lima et al., and Sartor et al. ,studied determinants of change of PSA over time and its association with recurrence following external beam radiation therapy of PC in 5 large cohorts (1, 4). 

They used a linear mixed model (LMM) for predicting PSA evolution and incorporated it to Cox model for risk of clinical recurrence and developed joint latent class model. Tsiatis and Davidian proposed a conditional score and semi-parametric likelihood based approach for the situation where the usual normality assumption is relaxed (12). The aim of this study was to use the joint modeling to evaluate the relationship between the longitudinal PSA data and PC recurrence.

## Methods

This historical cohort study was performed on 422 PC patients after its approval by the Ethics Committee of Mazandaran University of Medical Sciences. Inclusion criteria were: patients with localized PC and referring to Cancer Institute in Tehran (Iran) from 2007 to 2012 and those who underwent with radiation therapy.

Excluding criteria were: 1) another type of cancer, 2) no pre- or post RT prostatectomy, 3) the existing of data on covariate variables and 4) The completion of treatment follow-up period. Based on the inclusion and exclusion criteria, 314 patients were selected for the study. All patients diagnosed with PC, underwent radiation therapy. And then for each visit, PSA in blood serum was measured and recurrence or metastasis status was recorded. In patients with recurrence or metastasis, the onset time was recorded, but for the other patients it was declared as censored. Longitudinal measurements of PSA have transformed by:

for abnormal distribution reason. PSA* was observed measurement.

**Joint Model: **The most common method for joint modeling longitudinal and survival data is Cox proportional hazards model (13). But in some situations, there is deviation of assumptions for Cox model and it is not appropriate. In recent years, many different joint models have been suggested and different estimation methods were proposed by investigators (8-12, 14-15). The random effects model on the repeated measures of PSA as the longitudinal sub model, were used as follows:

$m_{i}\left(t\right)=x_{i}^{T}\left(t\right)\beta +Z_{i}^{T}\left(t\right)b_{i}+\varepsilon_{i}\left(t\right)\varepsilon_{i}\left(t\right)\mathrm{\sim N}(0,\sigma^{2})$

Here is fixed effect design matrix and is random effect, and bi are their coefficients respectively. 

Malignant cells release high amount of PSA in blood, and PSA is a continuous marker of disease progression, thus PSA slope change is the most important factor for estimating PC recurrence. We computed PSA slope by derivative related to time which is shown by . Then a joint model was defined as below:

In this model, denotes the baseline hazard function and is coefficient of PSA real measurements associated with risk function, is coefficient for PSA slope, and is vector parameter for other exploratory variables. The Cox model parameters are estimated with the approximate partial likelihood, but it is not appropriate for random effect sub models in joint model (13). We used the three methods; piecewise-constant approach, regression spline approach and Weibull distribution for complete likelihood function, estimating and computing hazard function parameters. After comparing these methods via Akaike information criterion (AIC) and goodness of fit model, the Weibull method estimator was better than others (16). EM algorithm, Newton-Gauss and Gauss-Hermit law were used for final model parameters. R software Version 3.2 was used for statistical analysis (available from: http://www.r-project.org/) (17).

## Results

**Figure 1:**
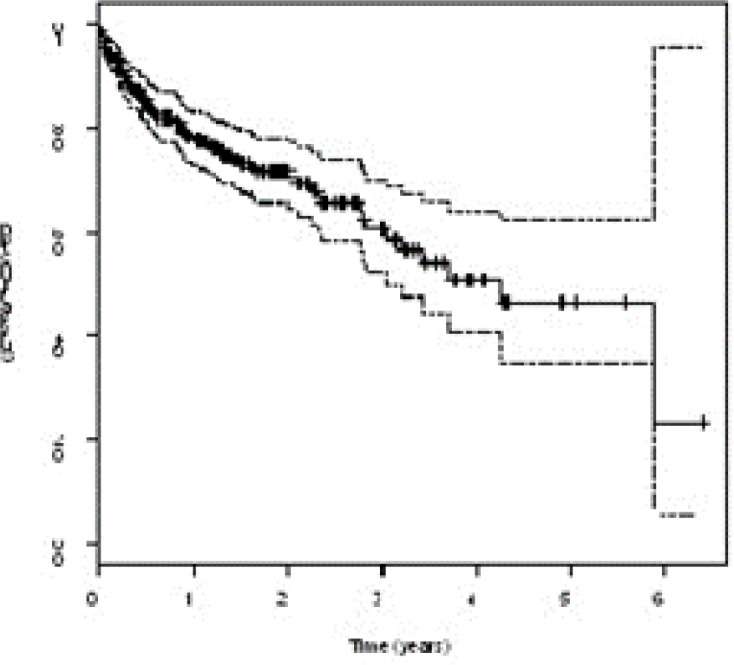
Kaplan-Meier estimate with 95% confidence bounds

In this study, based on the inclusion and exclusion criteria, out of 422 patients, data for 314 subjects were analyzed. Range of PSA values in 1184 tests was 1 to 20, mean number of PSA measures per patient was 3.77, median of pre-treatment PSA was 16 (ng/ml) and post-treatment was 6.35 (ng/ml). Median of total dose was 70 GY, median of Gleason score was 7. Demographic, pathological and clinical information are given in table1. In this study, 24.7 % cases had experienced recurrence or metastasis, 75.3 % were reported right censor (no event) on study duration. Survival cure median was 4.26 years (95% CI: 3.17- 5.34). Nonparametric Kaplan-Mayer estimation is show in figure 1. 

**Table 1:** Pathological, demographic and clinical characteristics of patients

Characteristics		Descriptive Statistics
**PSA (ng/ml; median (5th, 95th centile))**		6.35(0.1,108)
**Initial PSA (ng/ml; median (5th, ** **95th centile))**		16(1.145,150)
**Age at RT, years; median (Interquartile Range)**		71 (56.75,80)
**Gleason score (Number (%))**	2-5	41 (13.1)
	6	65 (20.7)
	7	125 (39.8)
	8-10	83 (26.4)
**Stage (number, (%))**	1	63 (20.1)
	2	164 (52.2)
	3 or 4	87 (27.7)
**Total dose (Gy; median, (5th, 95th centile))**		70 (30,72)
**Average dose per fraction (Gy; median, (5th, 95th centile))**		2 (2,3)
**Overall treatment days (median (5th,95th centile))**		35 (10,36)
**Number of follow-up PSA tests**		1184
**Years of follow-up (median (5th, 95th centile))**		0.87(0.04,3.82)

Joint model has two components or sub-models and we showed the results via a parameter estimating for longitudinal sub-model and proportional hazard sub-model. The main effect of hormone therapy, total dose and interaction between them via linear mixed model was displayed. Also a quadratic function for PSA trend by time was considered. Regression coefficients, standard deviation for variables and significant level are displayed in table 2. Hormone therapy was not statistically significant, but its interaction with time had significant association on longitudinal sub-model. Proportional sub-model analysis indicated he significant association of t-stage on PC recurrence risk. Therefore, patients with stage 3 or 4 have 5.79 times more recurrence risk than the other patients. 

Hormone therapy decreases recurrence risk by 42%, but not statistically significant. Patients who received total dose greater than 70 GY in comparison with other patients had lower recurrence risk, minimum 33% and maximum 87%. Age and Gleason score have no statistical significant on survival sub-model (table 3). The main result of joint model is displayed in table 4. PSA directly and significantly associated with recurrence risk, therefore increasing 2.6 ml/lit PSA (one unit in transformed PSA) increases 39% recurrence risk (95% CI for RR: 1.09-1.77). Also the slope of PSA trend has significant association with PC recurrence risk (95% CI for RR: 1.05-1.41).

**Table 2:** Longitudinal sub-model

Factors	Regression coefficient	Standard deviation	Confidence interval		p-value
			lower	upper	
**Intercept**	3.67	0.24	3.19	4.14	<0.001
**Hormone therapy**	-0.21	0.20	-0.61	0.18	0.295
**Total dose radiation (51-69** **Gy)**	-1.45	0.25	-1.93	-0.97	<0.001
**Total dose radiation(70< Gy)**	-1.39	0.20	-1.79	-0.99	<0.001
**Time (year)**	-1.48	0.27	-2.01	-0.95	<0.001
Time*hormone therapy	-0.58	0.24	-1.04	-0.11	0.015
Time* total dose	0.63	0.17	0.29	0.96	<0.001
Time * total dose (70< Gy)	0.19	0.15	-0.11	0.49	0.227

**Table 3:** Survival sub-model for hazard function with Weibul distribution

Factors	RR	Confidence interval		p-value
		lower	Upper	
**Intercept**	0.12	0.01	1.72	0.117
**Hormone therapy**	0.58	0.30	1.09	0.092
**t-stage (III, IV)**	5.79	3.03	11.04	<0.001
**Total dose radiation (51-69)**	0.62	0.28	1.36	0.233
**Total dose radiation (70<)**	0.30	0.13	0.67	0.003
**Gleason score (6)**	0.66	0.22	2.00	0.460
**Gleason score (7)**	0.84	0.32	2.25	0.732
**Gleason score (8<)**	0.84	0.32	2.22	0.730
**Age**	1.02	0.99	1.05	0.301

**Table 4:** Joint model parameter estimate for association between longitudinal and survival

Parameter	Regression coefficient	Standard deviation	RR	Confidence interval		p-value
				lower	Upper	
α	0.33	0.12	1.39	1.09	1.77	0.008
$\alpha_{s}$	0.19	0.08	1.21	1.05	1.41	0.011

## Discussion

Awareness of the link between PSA and PC recurrences in patients who have been treated clinically is important, because PSA is directly affected by prostate tissue and it increases with increased prostate tumor antigen concentrations. This property of PSA enables physicians to examine the changes in prostate tumor status. Today, with advances in statistical methods, it is possible to examine the relationship between internal covariate and recurrence risk.

In this study, the relationship parameters between two sub-models simultaneously were estimated by likelihood function. Based on the obtained results, there is a strong relationship between PSA and its slope with PC recurrence risk. The risk of recurrence significantly increased with the increase of both PSA and the PSA slope. Findings of Proust-Lima et al., (3) corresponded with our data, that is, they also found a correlation between PSA level and its slope with recurrence risk. Our study showed a clinical relapse in patients with stage 3-4 disease which agrees with the report of Taylor et al., (10, 11). In that study, PSA slope was significantly associated with relapse, but unlike our study, PSA levels are not significantly associated with the risk of relapse. Esfahani et al., (18) reviewed the literatures and selected a group of candidate biomarkers including PSA, sequential evaluation of PSA levels over time (PSA velocity), percentage free PSA and others biomarkers for the evaluation of PC prognosis. They found that PSAV, and ratio of increase of PSA before diagnosis, are the independent predictors of return of disease after radical prostatectomy. The diagnostic values of PSA velocity (PSAV), PSAV per initial volume (PSAVD) and PSA density (PSAD) were compared by Zheng et al., (19). PSAVD was a significant better indicator of PC than PSAV. Also the study of Anvar et al., showed that, the radiation dose and technique are associated with PSA slope and nadir, significantly (20). The study of Jackson et al., showed that a PSA doubling time of <6 months is prognostic for metastasis and prostate cancer-specific death (21). It means that the change of PSA slope is associated with disease recurrence, which agrees with our findings. In Taylor's study, hormone therapy was significantly associated with disease risk, in contrast to our data. This study showed relationship between PSA and its slope with the recurrence risk of PC with regard to the pathological, demographic and clinical features in the Iranian population. The results clearly indicated the link between these two PSA data longitudinal sub-model and recurrence risk sub-model by joint model in patients who underwent radiotherapy.
